# An Update on Venous Thromboembolism Rates and Prophylaxis in Hip and Knee Arthroplasty in 2020

**DOI:** 10.3390/medicina56090416

**Published:** 2020-08-19

**Authors:** Daniel C. Santana, Ahmed K. Emara, Melissa N. Orr, Alison K. Klika, Carlos A. Higuera, Viktor E. Krebs, Robert M. Molloy, Nicolas S. Piuzzi

**Affiliations:** 1 Department of Orthopaedic Surgery, Cleveland Clinic, Cleveland, OH 44195, USA; santand2@ccf.org (D.C.S.); emaraa2@ccf.org (A.K.E.); orrm2@ccf.org (M.N.O.); klikaa@ccf.org (A.K.K.); krebsv@ccf.org (V.E.K.); molloyr@ccf.org (R.M.M.); 2 Department of Orthopaedic Surgery, Cleveland Clinic Florida, Weston, FL 33331, USA; higuerc@ccf.org

**Keywords:** arthroplasty, joint replacement, venous thromboembolism, DVT, pulmonary embolism, surgical complications

## Abstract

Patients undergoing total hip and knee arthroplasty are at high risk for venous thromboembolism (VTE) with an incidence of approximately 0.6–1.5%. Given the high volume of these operations, with approximately one million performed annually in the U.S., the rate of VTE represents a large absolute number of patients. The rate of VTE after total hip arthroplasty has been stable over the past decade, although there has been a slight reduction in the rate of deep venous thrombosis (DVT), but not pulmonary embolism (PE), after total knee arthroplasty. Over this time, there has been significant research into the optimal choice of pharmacologic VTE prophylaxis for individual patients, with the objective to reduce the rate of VTE while minimizing adverse side effects such as bleeding. Recently, aspirin has emerged as a promising prophylactic agent for patients undergoing arthroplasty due to its similar efficacy and good safety profile compared to other pharmacologic agents. However, there is no evidence to date that clearly demonstrates the superiority of any given prophylactic agent. Therefore, this review discusses (1) the current prevalence and trends in VTE after total hip and knee arthroplasty and (2) provides an update on pharmacologic VTE prophylaxis in regard to aspirin usage.

## 1. Introduction

Total hip arthroplasty (THA) and total knee arthroplasty (TKA) are common and effective procedures for treating end-stage osteoarthritis, with approximately one million procedures performed annually and more than seven million people living with joint replacements in the U.S. [1,2,3,4,5]. The complication rate for these elective operations is low, but major complications such as venous thromboembolism (VTE) can be highly debilitating due to increased length of stay, potentially worsened outcomes or mortality, and an additional cost of $15,000–30,000 per episode [6,7,8,9]. Compared to other major operations, VTE occurs at a higher rate in THA and TKA, thus pharmacologic prophylaxis for VTE is largely considered to be necessary in these procedures [10].

Given the significant burden of VTE in THA and TKA there has been considerable effort directed towards prevention, and we would expect that the rate of VTE should diminish over time. Understanding the current rate and trends in VTE is important in allowing clinicians to evaluate the overall success of recent interventions and guide future studies in this field. Further, given the numerous means of multimodal and pharmacologic prophylaxis for VTE available, it is important to have a concise guide of contemporary research in this area. This review assesses the rates of VTE in THA and TKA and provides an update on pharmacologic VTE prophylaxis with a focus on the use of aspirin.

## 2. Overview of Venous Thromboembolism

VTE comprises both deep venous thrombosis (DVT) and pulmonary embolism (PE). DVT typically presents with pain, swelling, warmth, or erythema of the affected limb, most commonly occurring in the lower extremity, though it can occur in the upper extremity [11,12]. DVT may progress to PE, where a venous clot separates from its site of origin and becomes lodged in the pulmonary vasculature, and can be fatal.

### Pathophysiology of VTE in Total Joint Arthroplasty

The risk factors for VTE are described by Virchow’s triad: venous stasis, endothelial injury, and a hypercoagulable state. Two or more factors are generally necessary for the development of a VTE [13,14].

Venous stasis occurs both during surgery, because of tourniquet use (frequent during TKA) and intraoperative immobilization, and after surgery, when patients are less mobile in the post-operative period. This is especially likely to occur after THA and TKA when weight bearing joints have been reconstructed. Prolonged immobility is a risk factor for the development of VTE [15]. By supporting blood flow in the limbs, graduated compression stockings reduce the rate of DVT by approximately 57% after THA [16], and may reduce risk further when used while inpatient and at home [17]. There is also evidence that pre-operative common iliac vein compression increases VTE risk after THA, and that the choice of anesthesia may impact VTE risk for various reasons [18,19]. Rapid recovery programs and early mobilization may reduce the immobilization period after THA and TKA.

Endothelial injury is inevitable in surgery as a result of tissue dissection and manipulation, with a correlation between the extent of venous injury and the rate of VTE [20]. Although unavoidable, endothelial injury can be minimized through proper surgical techniques or minimally invasive techniques.

Hypercoagulable state. Finally, the local and systemic inflammatory responses induced by tissue injury during surgery lead to a hypercoagulable state. Elevations in pro-thrombotic molecules such as IL-6, C-reactive protein (CRP), and TNF-alpha are observed post-operatively [21,22]. Cellular injury causes the release of nucleic acids and histones [23]. Together, these molecules can trigger tissue factor (TF) and thrombin expression, the formation of neutrophil extracellular traps, and platelet activation, subsequently initiating the clotting cascade and thrombosis [23,24,25]. In addition, some patients may be predisposed to platelet hyperreactivity, further increasing their risk [26]. Stasis can contribute to hypercoagulability by increasing the concentration of pro-thrombotic factors at the sites of injury [27]. The use of polymethylmethacrylate (PMMA) cement further exacerbates the hypercoagulable state after orthopedic surgery as unreacted methylmethacrylate monomers may activate coagulation [23].

The importance of multimodal and pharmacologic prophylaxis against VTE by inhibiting the coagulation cascade is therefore evident. Indeed, the rate of DVT after THA without pharmacologic prophylaxis has been reported to be as high as 50% [28].

## 3. Rates of VTE after Total Hip and Knee Arthroplasty

### 3.1. Background

It has long been recognized that patients undergoing surgery are at high risk for VTE, but it was not until the 1960s to 1970s that pharmacologic VTE prophylaxis was employed. At that time, death from VTE afflicted approximately 1% of patients undergoing major surgery, with a DVT rate of approximately 20% [29,30]. After the introduction of heparin for VTE prophylaxis, the mortality rate declined to less than 1%, and the DVT rate to 3.6% [29,30].

The introduction of pharmacologic VTE prophylaxis was similarly effective in THA, as in other major operations. Morris et al. performed a randomized trial using heparin after THA and found the rate of venography screened DVT decreased from 50% to 11% [28]. Pellegrini et al. reviewed patients undergoing THA between 1984 and 1992, when warfarin or heparin were the main chemoprophylactic agents, and found a 1.2% rate of symptomatic DVT and 0.4% rate of symptomatic PE within 6 months [31]. Fender et al. found a 0.19% mortality rate due to PE in patients undergoing THA in 1990 [32].

Since that time, changes in VTE rates after arthroplasty have been more subtle, making it more difficult to correlate changes with individual interventions. Importantly, as the rates of VTE decreased, balancing the benefits of various prophylactic agents with the risks of adverse events has become a topic of increased interest. There have been countless studies on rates of VTE and prophylactic agents varying in methods, from retrospective, single-institution reviews to large randomized controlled trials. Therefore, we will discuss the recent literature assessing rates of VTE and chemoprophylactic approaches for THA and TKA focusing on prospective, large database, or meta-analysis study designs because of their better generalizability compared to regional retrospective reviews.

### 3.2. Methodologic Differences in Studying VTE

The reported rate of VTE depends significantly on how the outcome is defined, recorded, and studied. Routine post-operative screening for DVT reveals a large number of asymptomatic cases, inflating the VTE rate compared to the symptomatic rate [31]. Studies vary in how long after surgery VTE is measured, typically reporting in-hospital, 30-day, or 90-day rates. There is also discordance in rates of VTE between large regional registries and hospital administrative databases [33]. Depending on the setting, some hospital administrative databases have poor sensitivity for detecting VTE [34,35]. Finally, there may be differences in studies depending on whether or not they are industry funded [36]. These factors must always be considered while comparing rates of VTE after arthroplasty between regions or even between studies in the same region.

### 3.3. Recent Literature

#### 3.3.1. Primary Total Hip Arthroplasty

**Figure 1:**
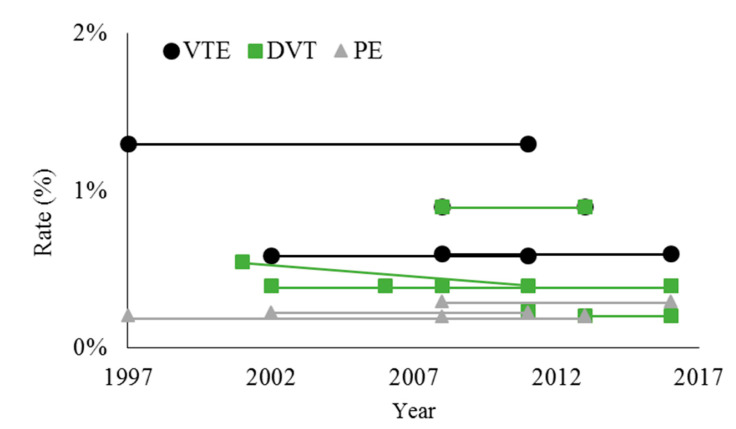
Overall rates of venous thromboembolism (VTE), deep venous thrombosis (DVT), and pulmonary embolism (PE) after total hip arthroplasty. Data points represent values reported by individual studies at the start and end of the study period. When there was no statistically significant difference over time, the average value is shown for both the beginning and end of the study period. References: [6,9,37,38,39,42,43,44].

The VTE rates after THA have remained relatively stable over the last two decades, though there is evidence of a decline in DVT rates, but not PE rates, prior to 2011 (Figure 1). Dua et al. used the U.S. National Inpatient Sample (NIS) to show that the rate of in-hospital DVT decreased from 0.55% in 2001 to 0.24% in 2011 [37]. Shahi et al. studied this same database from 2002–2011 and found an in-hospital VTE rate of 0.59% after THA (DVT 0.4%, PE 0.23%), and concluded that the rate of DVT but not PE declined over this time period [6]. Lieberman et al. performed a meta-analysis of randomized controlled trials from 1997 to 2013, and found the rate of PE after THA remained stable at 0.21% [38].

Further studies reporting on VTE rates prior to 2011 have largely corroborated these figures, though there has been regional variability in VTE rates that is only partially explainable. Pedersen et al. studied the Danish Hip Arthroplasty registry between 1997–2011 and found a 90-day VTE rate of 1.3%, with no significant change over this time period [39]. Lee et al. used the Korean National Health Insurance (NHI) claims database in 2010 and found a 90-day rate of VTE of 3.9% (DVT 2.7%, PE 1.5%), which is higher than other studies, possibly because the rate of chemoprophylaxis was only 37.3% [40]. Januel et al. studied VTE rates prior to discharge in 5 different countries (Canada, France, New Zealand, Switzerland, U.S. State of California) between 2006–2010 (dependent on the country) and found that the rate varied widely between 0.16–1.41% (DVT 0.07–1.15%, PE 0.08–0.46%) [41]. The variability was partially explainable by differences in lengths of stay and different rates of screening by ultrasound across countries.

Other studies have reported VTE rates for surgeries as recent as 2016, with no clear evidence that rates have declined. Warren et al. (2020) used the American College of Surgeons National Surgical Quality Improvement Program (NSQIP) database between 2008 and 2016 and found an average 30-day VTE rate of 0.6% (DVT 0.4%, PE 0.3%) [9]. The VTE rate in 2008 was 0.7% (DVT 0.6%, PE 0.2%) and in 2016 was 0.6% (DVT 0.4%, PE 0.3%), but there was no significant difference over time by multivariate logistic regression. Grosso et al. also studied the NSQIP database, and found a DVT rate of 0.41% between 2006–2016, with no change over that time [42]. Fuji et al. used an administrative database of Japanese patients between 2008 and 2013, and found an overall VTE rate of 0.9% (DVT 0.9%, PE 0.2%) [43]. Zeng et al. studied 78 hospitals in Asia between 2013–2016 and found an in-hospital DVT rate of 0.21% [44].

#### 3.3.2. Revision Hip Arthroplasty

Compared to primary THA, patients undergoing revision surgery are typically at higher risk of VTE. Warren et al. 2019 studied patients undergoing revision THA in the NSQIP database between 2008–2016 and found an overall VTE rate of 1.0% (DVT 0.7%, PE 0.4%) with no significant changes over the study period [8]. This is compared to a 0.6% VTE rate for primary THA over this same time period in the same database [9]. Courtney et al. queried the NSQIP database between 2011–2014 and found a slightly higher unadjusted risk of DVT for revision surgery (0.6% compared to 0.4% for primary), but no difference after controlling for confounding factors [45]. Shahi et al. studied the NIS from 2002–2011 and found an in-hospital VTE rate of 1.36% after revision THA (DVT 1.06%, PE 0.37%) with no significant decline over this time period [6].

#### 3.3.3. Primary Total Knee Arthroplasty

**Figure 2:**
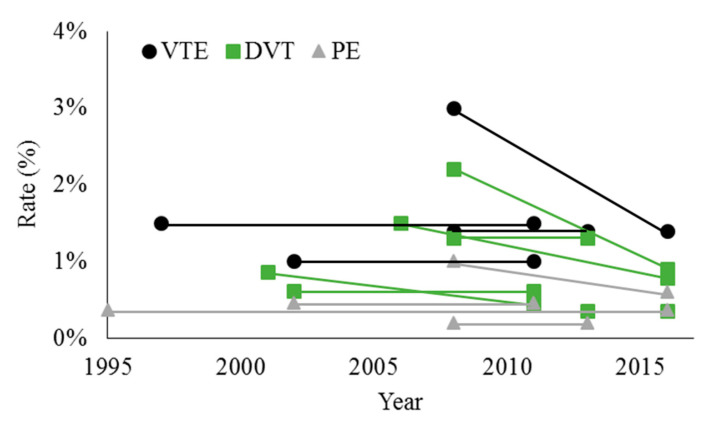
Overall rates of venous thromboembolism (VTE), deep venous thrombosis (DVT), and pulmonary embolism (PE) after total knee arthroplasty. Data points represent values reported by individual studies at the start and end of the study period. When there was no statistically significant difference over time, the average value is shown for both the beginning and end of the study period. References: [6,9,37,39,43,44,47,48].

TKA is more thrombogenic than THA and typically has a slightly higher rate of VTE [46]. However, there is evidence that the DVT rate after TKA has declined in the U.S. over the past two decades, yet the PE rate appears to have remained stable (Figure 2). Dua et al. used the NIS to show that the rate of DVT after TKA decreased from 0.86% in 2001 to 0.45% in 2011 [37]. Shahi et al. studied the NIS from 2002–2011 and found an in-hospital VTE rate of 1.03% (DVT 0.62%, PE 0.46%), and concluded that the rate of DVT but not PE declined over this time period [6]. 

Studies of TKA up to 2016 have largely demonstrated a further decline in the rate of DVT. Sarpong et al. used the NSQIP database between 2006–2016 to show that the 30-day DVT rate after TKA was 0.87%, with a decrease from 1.5% between 2006–2009 to 0.79% between 2014–2016 [47]. Warren et al. 2020 also used the NSQIP database between 2008 and 2016, and they found an overall 30-day VTE rate of 1.4% (DVT rate of 0.9%, PE rate of 0.6%) [9]. The VTE rate declined from 3.0% (DVT 2.2%, PE 1.0%) in 2008 to 1.4% (DVT 0.9%, PE 0.6%) in 2016, which was a significant change over time. One limitation of this study was that there were fewer cases of TKA in 2008 compared to that in 2016 (2668 versus 58,978), which could represent a selection bias.

Despite a decline in the rate of DVT after TKA performed in the U.S., rates of PE appear to have remained stable. Cote et al. performed a meta-analysis of studies between 1995–2016 reporting rates of PE after TKA, and demonstrated an average rate of 0.37% without a significant reduction over this time period [48].

Outside of the U.S., reported rates of VTE have been more variable, without clear evidence of a change over time. Pedersen et al. studied the Danish Knee Arthroplasty registry between 1997–2011 and found a 90-day VTE rate of 1.5%, with no significant change over this time period [39]. Fuji et al. used an administrative database of Japanese patients between 2008 and 2013, and found an overall VTE rate of 1.4% (DVT 1.3%, PE 0.2%), consistent with the Danish study [43]. Lee et al. used the Korean National Health Insurance (NHI) claims database in 2010 to show a 90-day VTE rate of 3.8% (DVT 3.2%, PE 0.7%), which may be significantly higher than other studies because the rate of chemoprophylaxis was only 48.4% [40]. Zeng et al. studied 78 hospitals in Asia between 2013–2016 and found an in-hospital DVT rate of 0.36%, which is much lower than rates in other studies [44].

Simultaneous bilateral TKA has been proposed as a treatment option for bilateral knee osteoarthritis, although concerns have been raised due to a higher risk of complications than in unilateral TKA [49,50]. Indeed, Masrouha et al. showed that the 30-day VTE rate after simultaneous bilateral TKA in the NSQIP database between 2008–2015 was greater than unilateral TKA (2.72% versus 1.45%), though there was no significant difference in mortality [51].

#### 3.3.4. Revision Knee Arthroplasty

Rates of DVT after revision TKA have likely decreased recently, with little evidence of a difference in VTE rates compared to primary TKA. Shahi et al. studied the NIS from 2002–2011 and demonstrated an in-hospital VTE rate of 1.17% (DVT 0.88%, PE 0.34%), finding that the rate of DVT but not PE declined over this time period [6]. In this same study, the VTE rate for primary TKA was 1.03%. Boylan et al. used the New York Statewide Planning and Research Cooperative System database between 2003–2012 and found a 90-day VTE rate of 2.13% (DVT 1.66%, PE 0.63%) for revision TKA with an odds ratio of 0.87 compared to primary TKA, suggesting that revision TKA could actually place patients at lower risk [52]. Warren et al. 2019 studied patients undergoing revision TKA in the NSQIP database between 2008–2016 and found an overall VTE rate of 1.2% (DVT 0.9%, PE 0.4%) with no significant changes over the study period [8]. This is compared to a 1.4% VTE rate for primary TKA over this same time period in the same database [9].

### 3.4. Bleeding and Mortality Following Knee and Hip Arthroplasty

The evidence reviewed thus far suggests that there has been a slight decrease in the rate of DVT after arthroplasty over the past one to two decades. Numerous factors may account for this reduction, but it is critical to compare rates of VTE to rates of bleeding in order to determine if we are simply trading-off complications with current VTE prevention strategies. A systematic review of randomized controlled trials compared rates of VTE and bleeding using low molecular weight heparin (LMWH) for VTE prophylaxis to rates with fondaparinux, rivaroxaban, dabigatran, or apixaban after THA or TKA [53]. This study found an overall rate of VTE of 0.99%, which is similar to the rates reported by the studies reviewed in this work. The rate of post-operative bleeding was 3.44%, more than three times the rate of VTE, suggesting that there is a tradeoff occurring between VTE prevention and bleeding. Orthopedic surgeons have raised concern that the potential increase in bleeding with the aggressive use of anticoagulation for VTE prevention can contribute to a delay in wound healing and increased infection risk. Overall, the increased risk of bleeding must be weighed with the benefits of anticoagulation for VTE prevention.

Although VTE poses significant morbidity to patients, it is important to understand whether reductions in VTE over time have correlated with reduced mortality. Xu et al. performed a pooled meta-analysis of THA and TKA for studies from 1950–2016 reporting post-operative mortality [54]. They show that prior to 1980, the mortality rate was 1.15%, and it decreased to 0.67% between 1996–2000. From 2001–2005, 2006–2010, and after 2011, the mortality rate continued to decline to 0.44%, 0.40%, and 0.24%, respectively. They further showed that mortality rates declined for subsets of patients receiving each of the common pharmacologic VTE prophylaxis agents, as well as those receiving no chemoprophylaxis, suggesting that factors other than these medications played a role in mortality reduction.

Compared to primary arthroplasty, revision arthroplasty may have higher mortality rates due to increased morbidity of the procedure. Using the NSQIP database, Warren et al. (2020) found an overall mortality rate of 0.2% for primary THA and 0.1% for primary TKA [9]. In the same database, the mortality rate was 0.7% for revision THA and 0.5% for revision TKA, higher than in the primary surgeries, though no statistical comparison between these figures has been made [8].

## 4. Pharmacologic VTE Prophylaxis

### 4.1. Guidelines

The most commonly used guidelines for VTE prophylaxis after arthroplasty are the American College of Chest Physicians (ACCP) guidelines (2012) [55] and American Academy of Orthopaedic Surgeons (AAOS) guidelines (2011) [56]. ACCP recommends pharmacologic prophylaxis be given for THA and TKA, with a Grade 1B recommendation for low molecular weight heparin, fondaparinux, dabigatran, apixaban, rivaroxaban, unfractionated heparin, vitamin K antagonists, and aspirin for a minimum of 10–14 days and up to 35 days. They suggest that low molecular weight heparin be used over the other agents (Grade 2C/2B recommendation). AAOS recommends the use of pharmacologic prophylaxis for both THA and TKA so long as patients are not at elevated risk for bleeding, but does not recommend any specific agents. Overall, orthopedic surgeons have to balance the increased risks of adverse events that stronger pharmacologic agents impose against the benefits prophylaxis given the individual risk factors for VTE of each patient.

One criticism of current guidelines is that they are unable to provide recommendations at the individual patient level. Globally, provider adherence to guidelines is relatively poor but has been improving; it has been suggested that adherence may be able to improve further if guidelines are able to provide advice on the individual patient level [57,58].

### 4.2. Prescription Patterns

There has been a trend towards increasing aspirin use for VTE prophylaxis. In a U.S. database between 2014–2016, 48% of THA cases and 43% of TKA cases received VTE prophylaxis consisting of a combination of aspirin and/or sequential compression devices (SCDs), while the remainder received one of the other pharmacologic agents [59]. In an Australian survey, the use of pharmacologic agents other than aspirin decreased from approximately 75% in 2012 to 40% in 2017 [60]. In a Korean database between 2008–2012, the most commonly prescribed agents for THA were LMWH (34%), heparin (23%), aspirin (19%), warfarin (14%), and novel oral anticoagulants (NOACs) (13%) and for TKA were LMWH (28%), NOACs (22%), aspirin (17%), warfarin (10%), and heparin (9%) [61]. In the Korean population from 2012–2013, only approximately 66–75% of cases of THA or TKA cases received pharmacologic prophylaxis [61,62,63]. Although many patients benefit from chemoprophylaxis, there is limited evidence that appropriately selected Asian patients may have a low rate of VTE after TKA without prophylaxis [64,65].

### 4.3. Aspirin for VTE Prophylaxis

The increasing use of aspirin for VTE prophylaxis has come with increasing evidence of its efficacy and safety. Matharu et al. performed a meta-analysis of 13 randomized controlled trials of aspirin for VTE prophylaxis and found a relative risk of 1.12 (95% confidence interval 0.79–1.62) compared to other pharmacologic regimens [66]. There was no statistically significant difference in individual risks of DVT or PE for aspirin compared to the other agents. Patients receiving aspirin had low but reduced risks of bruising and lower extremity edema, and no difference in bleeding, infection, or mortality. The largest trial in this meta-analysis, performed by Anderson et al., used aspirin after an initial 5-days of rivaroxaban for all patients [67]. Another meta-analysis, comparing aspirin to low molecular weight heparin, also found no statistically significant difference in VTE, bleeding events, or mortality, but notes that the evidence is poor and a well-constructed randomized trial is required [68]. Other studies have identified that the use of aspirin for VTE prophylaxis is associated with a lower mortality rate due to cardiac-related causes [69]. Retrospective reviews have shown that even in high risk VTE scenarios, such as simultaneous bilateral TKA or high-risk patient groups, aspirin has been equally as effective or more effective than other pharmacologic agents [70,71].

The proper dose of aspirin is an important consideration for optimizing its use. A systematic review constructed a generalized linear mixed model to compare low-dose (<162 mg/day) to high-dose (>162 mg/day) aspirin, and found no statistically significant difference between doses in terms of rates of DVT, PE, 90-day mortality, or major bleeding [72]. Recent, large, retrospective reviews have supported this finding, showing that low dose (81 mg) aspirin and high dose (325 mg) aspirin have similar outcomes in both THA and TKA [73,74].

Finally, there has been research on variants of aspirin use and additional benefits of aspirin. A randomized trial of aspirin in combination with fish oil found no statistically significant difference between this regimen and rivaroxaban for VTE prevention, but that it had lower rates of bleeding events [75]. Aspirin may also reduce rates of heterotopic ossification after THA [76].

As noted, randomized controlled trials of aspirin to date have been sparse, and those performed have not used aspirin as a standalone chemoprophylactic agent. There are trials underway that will address these shortcomings, including a randomized crossover trial in an Australian population comparing aspirin to low molecular weight heparin [77], and a trial in the U.S. comparing aspirin, warfarin, and rivaroxaban [78].

### 4.4. Patient Adherence to Medication Regimens

The efficacy of any medication is largely reliant on patient adherence after leaving the hospital. Although patients trust their providers about taking anti-thrombotic medications after arthroplasty, once a day medications, either oral or injectable, have better patient adherence than twice a day medications [79,80]. Even still, adherence to regimens is generally poor, and there is opportunity for strategies to improve patient adherence [81,82,83]. Aspirin is a desirable agent from this perspective because it is an oral medication that can be dosed once daily.

## 5. Discussion

The rates of VTE after total hip and knee arthroplasty have been relatively stable over the past two decades, with perhaps slight reductions in rates of DVT. Given that these events have become relatively rare, the most common means of studying VTE is using large registries or administrative databases. These data sources have inherent weaknesses, the most important being their sensitivity for recording VTE. The large variability in rates of VTE between different data sources could represent regional variability, but may also represent different sensitivities of different sources. Regardless, there appears to be consensus that the absolute rates of VTE after total hip and knee arthroplasty are currently low.

The already low rates of VTE, without dramatic recent change despite continued research into optimal pharmacologic prophylaxis as well as other means of reducing VTE, raises the question of the most successful strategy for reducing rates of VTE further while not increasing risks of adverse events, such as bleeding. Given that rates of post-operative bleeding are approximately three times greater than rates of VTE, we must be cautious with the aggressive use of anticoagulants for VTE prophylaxis. We have identified two main areas to focus on moving forward: (1) the development of enhanced personalized prediction models, which might help to detect patients at elevated risk of VTE and enable clinicians to construct a prophylactic regimen that optimally weighs the individual risks and benefits [84] and (2) if we can conclude that we have found an effective and safe pharmacologic agent that works well in concert with other means of VTE prophylaxis, perhaps the most high yield strategy for reducing rates of VTE further would be to optimize both provider and patient adherence to these regimens. Indeed, a root cause analysis of VTE suggests that there are still VTE events that are avoidable with better adherence to pharmacologic prophylaxis guidelines [85]. Interventions aimed at improving adherence will require more work with individual patients and providers, but have the potential to minimize the already low rate of VTE after arthroplasty.

Overall, aspirin may be an appropriate first-line prophylactic agent for low-risk patients undergoing THA and TKA because evidence has thus far demonstrated that it is safe and effective, and it is a low-cost medication. Over the past decade, there has been significant experience using aspirin for VTE prophylaxis, with the evidence to date showing similar efficacy to other pharmacologic agents in appropriately selected patients. Importantly, aspirin has the potential to be a safer agent with fewer major bleeding events. Safety is an increasingly important metric when evaluating the appropriateness of different chemoprophylactic agents given the relatively low rates of VTE after arthroplasty. A recent review of the use of aspirin after THA and TKA also concludes that, for appropriately selected low-risk patients, aspirin following rivaroxaban (as performed in a recent randomized trial [67]) is likely safe and effective [86].

## 6. Conclusions

VTE has become a relatively infrequent complication of total hip and knee arthroplasty, with significant research being done regarding the optimal chemoprophylactic agents and regimens. The recent rates of VTE after arthroplasty have remained largely stable, with only a slight decrease in rates of DVT, but not PE, after arthroplasty in the last one to two decades. Concomitantly, the use of aspirin as a prophylactic agent has increased as growing evidence supports its safety and efficacy. Aspirin use in select patients is associated with a low incidence of complications and may carry additional benefits, such as a reduction in the rate of myocardial infarction. Given that rates of post-operative bleeding exceed those of VTE, it is important to find an effective multimodal approach (including pharmacologic agents) that minimizes bleeding risk. Although studies of aspirin to date have shown that it is as effective as more potent anti-coagulants in select patients, a rigorous determination of its efficacy and safety through randomized controlled trials is still needed. It is still necessary to tailor the VTE prophylaxis choice to the individual needs of each patient as part of a multimodal prevention strategy, and predictive decision-making tools might provide value to this process. Finally, there is opportunity to improve patient adherence to pharmacologic regimens and provider adherence to VTE prophylaxis guidelines.
